# Dyspepsia—Its Causes and Cure

**Published:** 1870-06

**Authors:** 


### ARTICLE X.

### *Dyspepsia—its Causes and Cure.*

Dyspepsia has too often been called an incurable disease, the opinion arising, no doubt, from a misappreciation of its causes, and the treatment necessary to its cure. Probably often, also, from the lack of patience on the part of the one afflicted, in the use of the remedies necessary to accomplish the desired end; for, be it remembered, time and patience will be tasked severely in removing the cause, ere the cure can be effected.

It is caused less frequently by over eating, or errors in diet then is generally supposed; it arises more frequently from irregularity in habits of body, as taking of meals, evacuating the bowels, etc.

Nervous disorders are perhaps the most prolific source of the evil, and these, in their turn are induced by over work, worry, confinement, bad air, and insufficient food. The remedies are obvious: plenty of sunlight, exercise in the open air, care being taken not to exercise so violently as to cause weariness, a generous diet, and plenty of sleep, which is emphatically food for nerves—more benefit being often derived from a short nap before dinner to rest the stomach, than much medication.

When the trouble arises from gastric irritability, as it often does, care must be exercised in suiting the diet to the enfeebled powers of the digestive apparatus.

Sub-nitrate of bismuth, in doses of from five to ten grains, after eating, has usually a very salutary effect. Tonics and mild stimulants are also indicated to increase the appetite, and give tonicity to the nervous system generally. Above all, let the dyspeptic take his meals at stated intervals, absolutely abstaining from all food between meals.

Let their be variety in his food, good beef, chicken, mutton, oysters, eggs, good light stale bread, white or brown as he may prefer. This, with just sufficient exercise to aid and not impair digestion by fatigue, will almost always relieve the worst forms of the disease. Care should be exercised also in regard to locality, as low, damp, or deeply shaded spots will inevitably tend to depression. Change of scene and air is also highly beneficial; traveling by sea or land will often effect a cure. The nervous dyspeptic will do well to acquire the habit of loafing, for, to him, rest is more beneficial than exercise. Sunlight is, however, the most powerful agent known, frequent sunbaths sweetening and invigorating both the invalid's temper and digestion.

General electrization has of late years become a very popular and efficient remedy, but requires time and perseverance to secure its effects, as, indeed, does every one applicable to the peculiar forms of this disease. Perhaps a more efficient aid to all remedies is the caution in regard to dress, especially among fashionable females. Gastric irritability and consequent weakness is sure to follow the cruel practice of compressing the waist, especially when the modern corset must be supplied with an inflexible support, the lower extremity of which presses directly upon the stomach, and acts as a source of constant irritability. The monstrous belt buckle, also, as unyielding as metal can make it, is a cunningly devised instrument of torture. The cure for this evil, of course, is simply to allow nature to fashion the bust, and be content therewith.

Dr. Baird gives the following rules to be observed by the dyspeptic: Never exercise hard before breakfast, nor, indeed, before or after any meal; take the exercise you most enjoy; never exercise to absolute exhaustion; to which might be added, never starve yourself—let your diet be light, but abundant.—*Oregon Med. and Surg. Journal.*

### MONTHLY SUMMARY.

